# Oral health related quality-of-life outcomes of partially edentulous patients treated with implant-supported single crowns or fixed partial dentures

**DOI:** 10.4317/jced.53661

**Published:** 2017-05-01

**Authors:** Bader K. AlZarea

**Affiliations:** 1 Associate Professor, College of dentistry, AlJouf University, Saudi Arabia

## Abstract

**Background:**

Oral health-related quality of life (OHRQoL) is afflicted by different variables. Limited information is available regarding the impact of different phases of implant therapy on OHRQoL of edentulous patients. This study was carried out to assess the OHRQoL of patients treated with implant-supported single crowns or fixed partial dentures.

**Material and Methods:**

A total of 79 healthy partially edentulous subjects needing implant therapy were incorporated in this study. Before placement of the implants, the subjects were instructed to fill the original version of OHIP questionnaire. Subsequently patients received titanium oral implants of the ITI® Dental Implant System. After 1st, 2nd and 3rd year of implant placement, patients filled the same OHIP-49 questionnaire. In this manner the impact of implant therapy on OHRQoL by putting in comparison pre- and post-treatment OHIP-49 scores was assessed. Statistical analyses were performed using Statistical Package for the Social Science software (SPSS, version 22, Chicago, IL, USA). Paired t test and Unpaired t test were performed and a statistical significance was set at 5% level of significance (*p*<0.05).

**Results:**

Functional limitation, physical pain, psychological discomfort, physical disability, psychological disability, social disability were significantly decreased from baseline to 1st year (*p*<0.05) except handicap (*p*>0.05). All variables were also significantly decreased from baseline to 2nd year and 3rd year (*p*<0.05). There were no significant differences dependent on gender with respect to OHIP (*p*>0.05). Patients aged less than 60 years and more than 60 years of age groups differed significantly with respect to OHIP scores measured at 1st year, 2nd year and at 3rd year of implant placement (*p*<0.05).

**Conclusions:**

Decrease in pre- and post-treatment OHIP scores OHIP demonstrated the significant increase in the OHRQoL after the therapy, which suggested increased levels of patient satisfaction.

** Key words:**Edentulism, dental implants, Oral health-related quality-of-life.

## Introduction

Edentulism is viewed as a physical disability since imperative body part has been lost and in the aged individual’s edentulism adversely affects distinctive parts of personal satisfaction. Numerous edentulous individuals experience a debilitated competence to perform vital life assignments (1).

Diverse treatment conceivable outcomes have been put forth to supplant the missing tooth. Till recent time, removable and fixed partial dentures were two principal choices for refurbishing the capacity and aesthetics. However as of late, implant therapy has achieved more attention and connotation. Implant therapy is considered as a compelling addendum to conventional fixed and removable and fixed prosthodontic treatment for replacing missing teeth (2).

Endurance of the implant, prosthesis durability, and the recurrence of complexities are viewed as the most noteworthy results for a prosthodontist, whereas social and psychological impacts of treatment, cost adequacy, advantage, and utility are more essential from the patient’s point of view (3,4).

Oral health influences the quality of life in greater part of individuals and the sort, nature of prosthodontic replacement, and recently all the more frequently dental implants, can be viewed as one part of oral health in aged individuals (5).

Oral health-related quality of life (OHRQoL) is an essential patient-focused endpoint to be considered while surveying the effect of alterations in the oral cavity and assessing proficient intercessions (6). OHRQoL is a more thorough, multidimensional assessment of oral diseases and prosthodontic therapy than patient gratification only. Even though various oral health-definitive estimations have been created in the course of the last two decades, the Oral Health Impact Profile (OHIP) has risen as an intense device in the appraisal of OHRQoL. A current exemplary of oral wellbeing was utilized by Locker to recognize applied realms in the order of social influence. In this model, ailment can prompt debilitation, then utilitarian impediment, and at last physical, psychologic, or social inability, depicted as any constraint in or the nonattendance of the capacity to perform day to day living activities (7,8).

In the overall populace, the count of teeth has the most grounded effect on the OHRQoL. Many studies have for the most part been centered around OHRQoL results of conventional prosthodontic therapy and few studies also assessed variation in OHRQoL after implant therapy (9).

Enhancement in quality of life is noted in individuals who were treated with implant supported removable overdentures in correlation with use of complete dentures. Implant-supported dentures catered more noteworthy change of oral health in terms of masticatory efficacy, solace, discourse, function, improved personal appearance. Changes in practical angles and oral well being have been confirmed in geriatric patients who were rehabilitated with implant prosthesis (10-14).

Several researches were carried out in the field of patient based results has for the most part been focused on implant-supported removable denture and very minimal data is available regarding outcomes with treatments such as implant supported single crowns or fixed partial dentures, (15). With this background, this study has been carried out with an objective to assess the OHR-QoL of patients treated with implant-supported single crowns or fixed partial dentures.

## Material and Methods

During the period from August 2013 to June 2016, all patients referred for implant therapy to the department of Prosthodontics, College of Dentistry, AlJouf University were selected using the following inclusion criteria:

• Compliant to participate in the whole time span of this research

• Sound general health condition

• Competent to discern and reciprocate to the questionnaire

• Satisfactory oral hygiene and no signs of soft or hard tissue inflammation 

• Sufficient bone volume to insert the implants

• Presence of one or more of the indications for placement of the implants (16).

Exclusion criteria for the study subjects were maintained in order to avoid bias for sampling and included:

• History of radiotherapy or chemotherapy, osteoporosis, or bisphosphonate therapy.

• Mental conditions that could affect a patient’s consistence to implant therapy and cooperation in the study

• Chronic and refractory periodontal disorders

• Subjects with bruxism and temporomandibular disorders

• Subjects taking anticoagulant therapies, with bleeding disorders, uncontrolled diabetes

• Pregnant and diabetic patients

According to these criteria, a total of 79 subjects were screened and incorporated in this study. All the subjects provided informed consent, which comprised of a complete discourse regarding the potential favorable circumstances and possible exasperation of the proposed implant therapy.

Before placement of the implants, the subjects were instructed to fill the original version of OHIP questionnaire with 49 questions developed by Slade and Spencer (17).

High OHIP scores demonstrated poor OHRQoL, albeit low OHIP scores represented gratifying and competent OHRQoL.

Subsequently patients received titanium oral implants of the ITI® Dental Implant System (Institut Straumann AG, CH4437 Waldenburg, Switzerland). The implants were placed according to the manufacturer’s instructions (18). The implants were restored by means of single crowns or fixed partial dentures. After 1st, 2nd and 3rd year of implant placement, patients completed again the same OHIP-49 questionnaire. Thusly the impact of implant therapy on OHRQoL by putting in comparison pre- and post-treatment OHIP-49 scores was assessed.

All statistical analyses were performed using Statistical Package for the Social Science software (SPSS, version 22, Chicago, IL, USA). Paired t test and Unpaired t test were performed (used to assess the differences between answers of participants) and a statistical significance was set at 5% level of significance (*p*<0.05).

## Results

Out of 79 samples, 47 were males with mean age of 46.1 ±10.18 and 32 were females with mean age of 43.5 ± 11.9. Similarly, a total of 232 implants were placed with mean 3.01. Out of 232 implants, a maximum of 34.91% and 34.05% of implants were placed respectively at premolar and molar teeth followed by 21.12% in incisors and 10.78% in canines. A maximum of 26.58% patients were having 3 implants as compared to 22.78% having 2 implants, 18.99% had one implants followed by a minimum of 6.33% patients having 6 implants.

The mean and SD before implant placement, 1st year, 2nd year, 3rd year are mentioned in  Table 1. From the results of the  Table 2 it can be seen that the variables, functional limitation, physical pain, psychological discomfort, physical disability, psychological disability, social disability were significantly decreased from baseline to after 1st year (*p*<0.05) except handicap (*p*>0.05). All variables were also significantly decreased from baseline to after 2nd year and 3rd year (*p*<0.05).

**Table 1:**
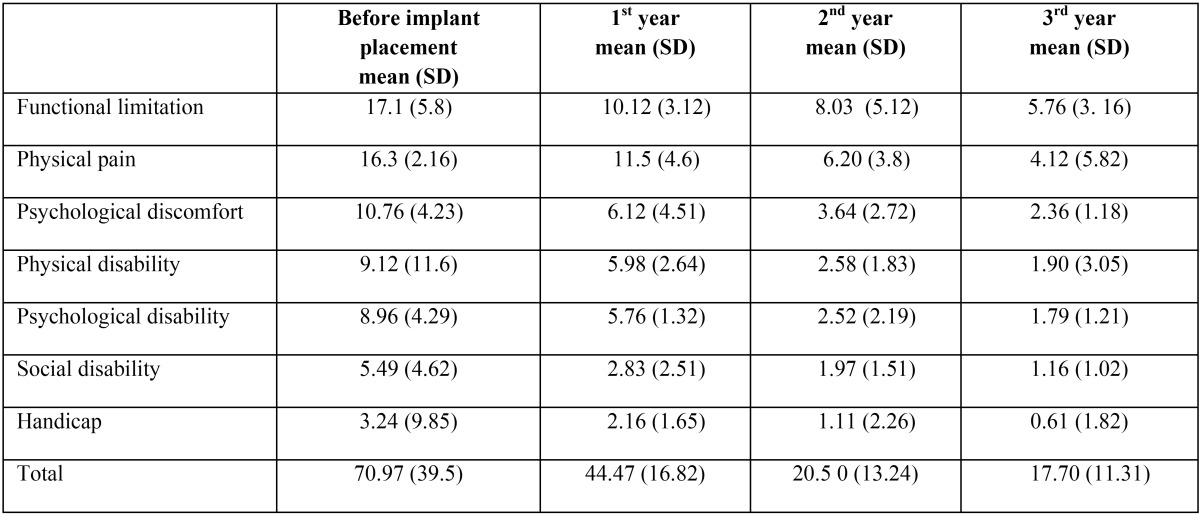
Comparison of mean OHIP scores (SD) in patients (Before implant placement, 1st year, 2nd year, 3rd year).

**Table 2:**
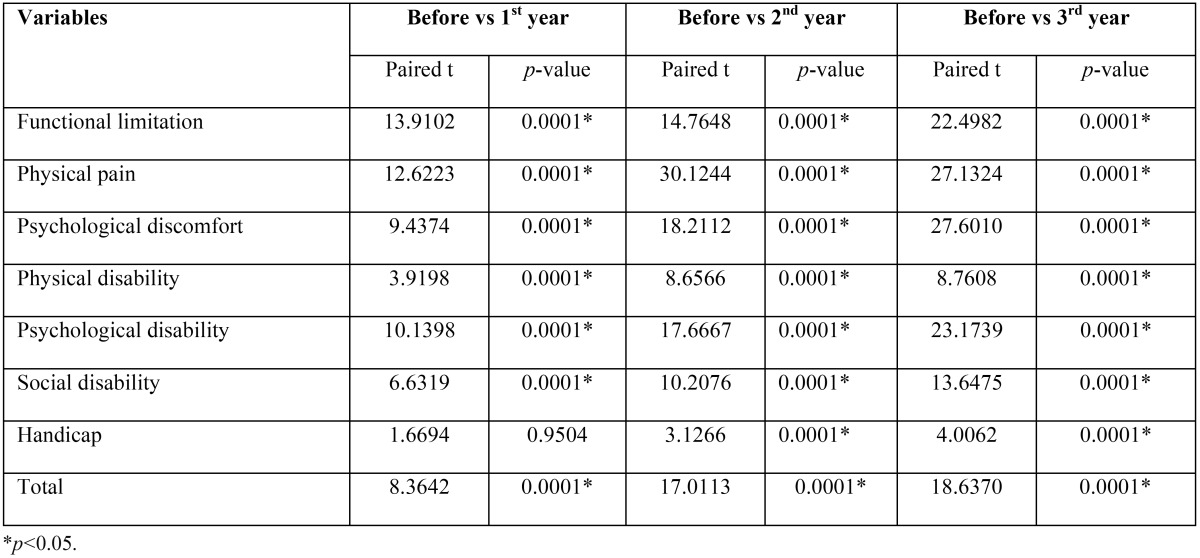
Comparison of mean OHIP scores (SD) in patients (Before implant placement, 1st year, 2nd year, 3rd year) by paired t test.

The male and female do not differ significantly with respect to OHIP scores measured at baseline, 1st year, 2nd year and 3rd year implant placement at 5% level (*p*>0.05) ( Table 3). Patients aged less than 60 years and more than 60 years did not differ significantly with respect to OHIP scores measured before implant placement at 5% level (t=0.5070, *p*>0.05). The patients less than 60 years and more than 60 years of age groups differed significantly with respect to OHIP scores measured at 1st year implant placement at 5% level (t=2.1525, *p*<0.05), 2nd year implant placement at 5% level (t=8.3138, *p*<0.05) and when measured at 3rd year implant placement at 5% level (t=3.0428, *p*<0.05) ( Table 4).

**Table 3:**
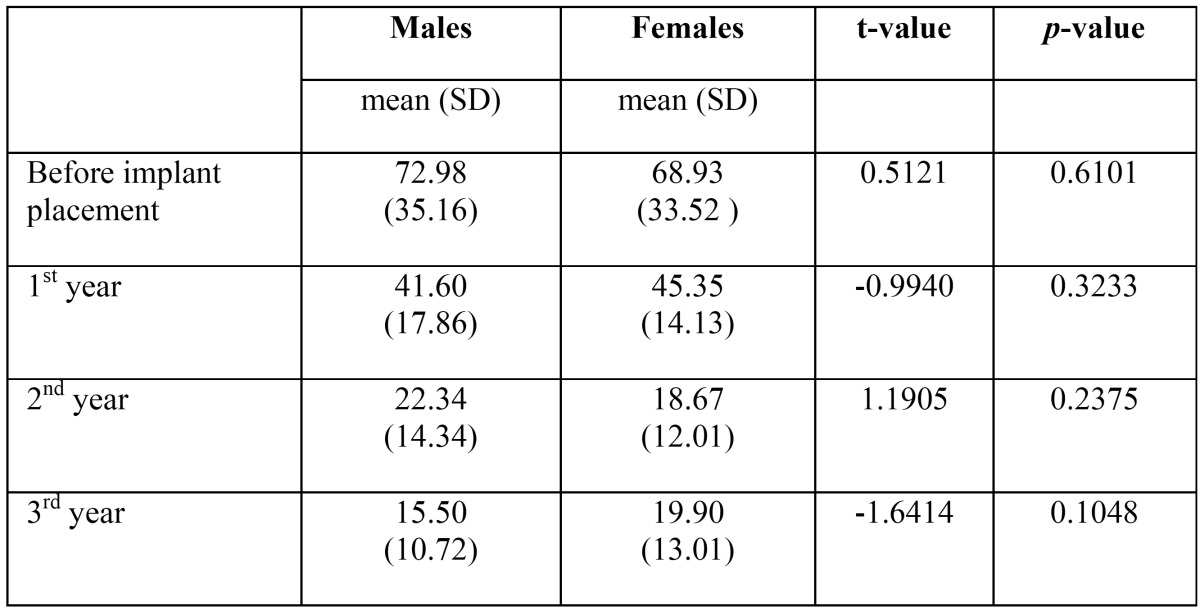
Mean (SD) OHIP scores measured at four occasions the improvement on the oral health related quality of life after the prosthetic treatment using the OHIP-14 by gender.

**Table 4:**
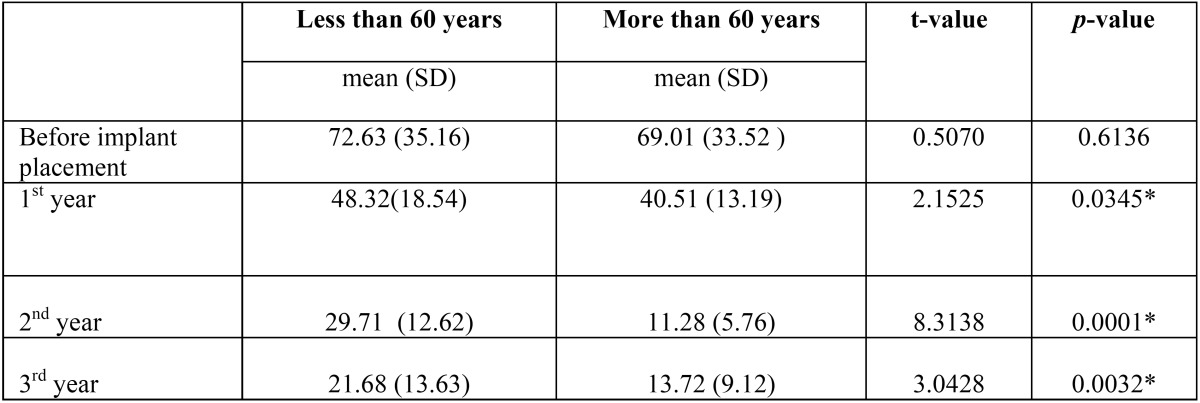
Improvement in the oral health related quality of life after the prosthetic treatment using the OHIP by age groups.

## Discussion

Numerous studies have demonstrated progress in OHRQoL in subjects who were treated with implant-supported prostheses compared to conventional dentures. Studies proved that individuals who were with implant-supported overdentures showed a higher level of satisfaction than those who were treated with conventional full dentures (19-21). Many studies were carried out among edentulous individuals with implant-supported overdentures or implant fixed full dentures which revealed a increased satisfaction with both therapeutic modalities (22-24). Subjects who were provided with a single implant prosthesis also revealed a high satisfactory rate (25,26).

In the present study, OHIP questionnaire was utilized to screen transition of the attributable to its affectability to recognize the effect of prosthodontic therapy and its extensive usage (27).

The OHIP questionnaire was administered at various perception periods different: prior to placement of implants, at 1st year, 2nd and 3rd year after the placement. The rationale behind examining the patients for a more drawn out period was attributable to the way that longitudinal estimations assess better the general accomplishment of prosthodontic therapy in correlation with the minimal time phase (28).

The pre- and post-treatment OHIP summary and subscale scores of patients demonstrated a significant decrease in OHIP scores, which described the significant increase in the OHRQoL after the therapy, which suggested increased levels of patient satisfaction. This observation was in accordance with previous studies, which suggested that placement of IFPDs improved quality of patients life (15,27,29,30). The time interval of one year is opted for first follow up after treatment as it is mentioned by Petricevic *et al.*, that a time period in weeks would be too short to completely demonstrate the benefit of the prosthodontic therapy (15).

As the participants of this study belonged to a wider age, ranging from 39 to 81 years, it was prudent to segregate the individuals into two groups to know the impact of age. No significant difference of OHIP scores related to age was observed before implants placement and the OHIP scores were significantly lower in patients older than 60 years in comparison with the younger group after 1st, 2nd and 3rd year of placement. This observation was similar to the findings of the Petricevic *et al.* who observed a significantly lower OHIP summary score in the older patients compared to the younger group in the patients with implants after 3 years (15). Similar to this, Kouppala *et al.* evaluated the oral health-related quality of life of patients with implants using OHIP 14 and found that patients younger than 65 years of age had higher mean OHIP-14 sum scores compared to the older age group which suggested that younger patients exhibited lower levels of satisfaction (31). The general population in the most youthful age were typically engaged in working life and needed to adapt to various social circumstances, and the requests of oral status may have been higher than the geriatric patients. It has been observed that older individuals were contended with not as much as perfect oral wellbeing and they have minimal quixotic desires of the treatment than their younger counterparts. In contrast to this Awad *et al.* found that in the younger individuals, OHIP scores were significantly better in the implant group compared to the group restored with conventional complete dentures (28).

No significant differences in OHIP scores between males and females was observed in this study, this was in agreement with the results of Petricevic *et al.* (15) Kouppala *et al.* (31) Strassburger *et al.* (32) and Baba *et al.* (33) in contrast to this Siadat *et al.* observed that patient satisfaction varied according to the gender (34).

Zitzmann and Marinello reported an improvement in function, psychologic well being, and self-esteem for patients who received removable or fixed implant prostheses but saw no significant difference between the two types of prostheses (35).

The OHIP was utilized as a measure of OHQOL for this research since it has great interior unwavering quality and has been approved in various cross-sectional populace contemplates. The utilization of an accepted questionnaire, for example, the OHIP encourages examinations with other comparative studies.

## Conclusions

The pre- and post-treatment OHIP summary and subscale scores of patients in the present study established a decrease in OHIP scores portraying the compelling increase in the OHRQoL after the therapy, which implied increased levels of patient satisfaction.
